# Advancements in Immunotherapeutic Treatments for Hepatocellular Carcinoma: Potential of Combination Therapies

**DOI:** 10.3390/ijms25136830

**Published:** 2024-06-21

**Authors:** Yusra Zarlashat, Hassan Mushtaq, Linh Pham, Wasim Abbas, Keisaku Sato

**Affiliations:** 1 Department of Biochemistry, Government College University Faisalabad, Faisalabad 38000, Pakistan; yusrazarlashat@gcuf.edu.pk; 2 Health Biotechnology Division, National Institute for Biotechnology and Genetic Engineering-C (NIBGE), Faisalabad 38000, Pakistan; hassanmushtaq245@gmail.com; 3 Pakistan Institute of Engineering and Applied Sciences (PIEAS), Islamabad 45650, Pakistan; 4 Department of Science and Mathematics, Texas A&M University-Central Texas, Killeen, TX 76549, USA; linhpham@tamuct.edu; 5 Department of Medicine, Division of Gastroenterology and Hepatology, Indiana University School of Medicine, Indianapolis, IN 46202, USA

**Keywords:** hepatocellular carcinoma, immunotherapy, tumor microenvironment, immune checkpoint inhibitors, combination therapy

## Abstract

Hepatocellular carcinoma (HCC) is the sixth most prevalent cancer and a significant global health burden, with increasing incidence rates and limited treatment options. Immunotherapy has become a promising approach due to its ability to affect the immune microenvironment and promote antitumor responses. The immune microenvironment performs an essential role in both the progression and the development of HCC, with different characteristics based on specific immune cells and etiological factors. Immune checkpoint inhibitors, including programmed death-1/programmed death-ligand 1 inhibitors (pembrolizumab, nivolumab, and durvalumab) and cytotoxic T lymphocyte antigen-4 inhibitors (tremelimumab and ipilimumab), have the potential to treat advanced HCC and overcome adverse effects, such as liver failure and chemoresistance. Phase II and phase III clinical trials highlight the efficacy of pembrolizumab and nivolumab, respectively, in advanced HCC patients, as demonstrated by their positive effects on overall survival and progression-free survival. Tremelimumab has exhibited modest response rates, though it does possess antiviral activity. Thus, it is still being investigated in ongoing clinical trials. Combination therapies with multiple drugs have demonstrated potential benefits in terms of survival and tumor response rates, improving patient outcomes compared to monotherapy, especially for advanced-stage HCC. This review addresses the clinical trials of immunotherapies for early-, intermediate-, and advanced-stage HCC. Additionally, it highlights how combination therapy can significantly enhance overall survival, progression-free survival, and objective response rate in advanced-stage HCC, where treatment options are limited.

## 1. Introduction

Hepatocellular carcinoma (HCC) is a liver cancer that arises from hepatocytes and accounts for 80% of all liver cancer cases [1]. As the most common primary liver cancer, HCC highlights the third major cause of cancer-related death globally, including all types of cancers [2], with five-year survival rates of 25.9–41.7% for early-stage, 5.9% for intermediate-stage, and 0.2–0.4% for advanced-stage HCC [3]. HCC is associated with various liver diseases, such as chronic viral hepatitis [4], liver cirrhosis [5], metabolic dysfunction-associated steatotic liver disease [6], and alcoholic steatohepatitis [7].

The risk factors linked to HCC include chronic hepatitis B virus (HBV)/hepatitis C virus (HCV) infection, alcohol drinking, tobacco smoking, and aflatoxins [8]. The incidence of HCC has been significantly increasing in recent years worldwide, and approximately 906,000 patients are diagnosed with HCC annually [9]. Men have a greater risk of developing HCC compared to women, with a ratio of approximately 2.8:1 [10]. In the late 20th century, several ablative methods including radiofrequency ablation (RFA), microwave ablation, and cryoablation were commonly performed to destroy tumors by either heating or cooling them, although these treatment options may not be useful for large tumors [11]. Chemotherapy is a standard therapy in which drugs are administered to target rapidly dividing cells and inhibit their growth [12]. Transarterial chemoembolization (TACE) is a chemotherapy technique that blocks tumor-supplying arteries by directly injecting embolic agents into the arteries that deliver 90% of the hepatic artery’s blood to the tumor [11]. Transarterial radioembolization (TARE) is an intra-arterial treatment that uses micro-sized radiation-carrying beads loaded with the radioactive compound Yttrium-90 to kill tumor cells [13].

The primary treatment options for HCC are surgical resection (full or partial hepatectomy) or liver transplantation [14]. However, the limited availability of liver donors and the increased incidence of HCC contribute to the restricted adoption of liver grafts [14]. Novel therapies are required since treatment approaches including chemotherapy, radiation, and ablation are limited by tumor size, side effects, and challenges at the advanced stage [15]. In the treatment of HCC, the main chemotherapeutics inhibit the multiple kinases involved in angiogenesis and tumor cell proliferation pathways by blocking receptor tyrosine kinases (RTKs), including vascular endothelial growth factor receptor (VEGF), platelet-derived growth factor receptor, fibroblast growth factor receptor, and mesenchymal–epithelial transition factor [16]. Conventional chemotherapeutics function either by intercalating into the DNA strands and forming crosslinks, or disrupting base-pair binding, which interferes with DNA replication and transcription processes, ultimately inducing apoptosis [17].

Accumulating evidence suggests that immunotherapy has the potential as a novel therapeutic strategy to cure HCC by providing a specific immunosuppressive environment to increase the survival rates of advanced-stage HCC patients [18]. HCC tumors are accompanied by dense stromal tissues called tumor microenvironments (TMEs), containing cancer-related fibroblasts, tumor cells, stromal cells, immune cells, and endothelial cells, which are all associated with HCC progression [19]. Immune checkpoint inhibitors (ICIs) are drugs targeting key coinhibitory signals to regulate immune cells in the cancer-immunity cycle [20]. An antitumor immunotherapy using anti-programmed death protein-1 (PD-1) and cytotoxic T lymphocyte antigen-4 (CTLA-4) antibodies demonstrated promising therapeutic effects for advanced HCC [21]. The antitumor activity of immune cells differs depending on tumor tissues and the TME [22]. The effectiveness of immune response is influenced by the TME composition, variations in tumor immunogenicity, and immunosuppressive factors [22]. This highlights the need for immunotherapeutic approaches that are specific to the unique immunological environment of HCC [23].

In a previous review, Rimassa et al. focused on the changes in systemic therapy for HCC, highlighting the importance of patient selection via the Barcelona Clinic Liver Cancer (BCLC) staging system and the development of molecular therapeutics like tyrosine kinase inhibitors (TKIs) and ICIs [24]. This review summarizes the potential of immunotherapy and combination treatments, highlighting the shift from cytotoxic chemotherapies to targeted treatments like sorafenib, with evidence from phase III trials showing improved survival and disease progression in advanced HCC [24]. Wei et al. covered advancements and challenges over the past decade in HCC immunotherapy, emphasizing immune evasion mechanisms and the exploration of immunotherapy approaches for HCC [25]. The authors described the use of ICIs for advanced-stage HCC, alongside the development of novel drugs and combination therapies, explained by insights into TME molecular pathways [25]. Stefanini et al. summarized the recent success and future directions of treatments using ICIs and TKIs, particularly highlighting atezolizumab plus bevacizumab [26]. This review article emphasized the evolving landscape of first-line and second-line therapies over the past five years, focusing on targeting angiogenesis and immune evasion [26].

In the current review, we seek to analyze the further potential of immunotherapies against HCC, especially the potential of combinations of ICIs with TKIs or anti-VEGF antibodies in clinical studies. We summarize stage-specific immunotherapy strategies and provide detailed clinical trial data, highlighting progression-free survival (PFS), overall survival (OS), and response rates. Additionally, this review addresses the mechanisms of ICIs, offering further insights into their therapeutic potential against HCC.

## 2. Literature Search Strategy

A literature search was conducted using PubMed, Google Scholar, Nature, and ClinicalTrials.gov, with keywords such as “HCC” or “HCC” in combination with “immune checkpoints”, “TME”, or specific drug names, such as “nivolumab”. These terms were used in various combinations to include a wide range of relevant studies, such as “HCC and TME”, “HCC and tislelizumab”, “early-stage HCC and neoadjuvant immunotherapy”, “advanced-stage HCC and pembrolizumab monotherapy”, “advanced-stage HCC and atezolizumab plus bevacizumab”, and “HCC and adoptive T cell therapy”. The literature search included many study types to ensure a broad understanding of the topic, including randomized controlled trials, clinical trials, pilot studies, and systematic reviews related to TKIs, ICIs, and anti-VEGF therapies for HCC. The inclusion criteria for this review were drugs used specifically for the treatment of HCC, such as durvalumab and tremelimumab, and others that have shown promising results. 

We focused on clinical trials and human studies to ensure the findings were directly relevant and applicable to clinical practice, specifically regarding the outcomes of immunotherapies for HCC. Both relevant preclinical and clinical trials with potential significance were considered, providing a comprehensive view of clinical insights. This highlights the necessity of clinical trials to directly assess potential benefits in human patients with HCC. Basic studies using animal models and in vitro studies using cultured HCC cell lines were excluded from this review because they did not fully replicate the complexity of human HCC and its TME to provide conclusive results, leading to potential differences in drug efficacy and safety. Studies of combination therapies for other cancers were not included, even if they showed promising performance, if no trials for HCC were available for those therapies. This review includes the most current and relevant findings in the rapidly evolving field of immunotherapy for HCC, focusing on articles published from 2017 to 2024.

## 3. Immune Checkpoint Inhibition in HCC

The hepatic cells in the TME include liver sinusoidal endothelial cells, hepatic stellate cells, macrophages or Kupffer cells, and various cells of the adaptive immune response, such as natural killer (NK) cells and cluster of differentiation 4^+^ (CD4^+^) or cluster of differentiation 8^+^ (CD8^+^) T-lymphocytes [27]. T-cell exhaustion in HCC is characterized by diminished proinflammatory responses, lower cytokine production, reduced proliferation, and impaired cytotoxicity [28]. Regulatory T cells (Treg) inhibit antitumor immunity, whereas CD8^+^ T cells can recognize and eliminate cancer cells, and CD4^+^ T cells regulate the activity of other immune cells [28]. Activated NK cells produce chemokines, cytokines, and cytotoxic granules that contribute to the death of the target tumor cells [29]. Dendritic cells (DCs) present tumor antigens to T cells to initiate strong immune responses, improving tumor cell recognition and targeted destruction [30].

Immune cells in the TME have a significant role in tumor progression [31]. It has been shown that tumor-associated immune cells may have either tumor-promoting or antitumor roles [31]. High populations of CD4^+^ and CD8^+^ T cells respond better to immunotherapy, providing an active and potentially effective antitumor immune microenvironment [32]. Although antitumor immune cells in the TME target and kill tumor cells in early tumorigenesis, tumor cells can undergo immune evasion and inhibit their cytotoxic effects through various mechanisms, e.g., the downregulation of antigen presentation, the intratumoral accumulation of immunosuppressive cell populations, the production of inhibitory cytokines, and the activation of multiple inhibitory receptor–ligand pathways [22]. Therefore, a high population of CD4^+^ and CD8^+^ T cells enhances the immune system, leading to improved patient outcomes and reduced tumor progression [22]. The overexpression of inhibitory immune checkpoint proteins on immune cells during tumor development is a crucial factor in cancer immunological escape and the suppression of antitumor immune responses [33]. Thus, inhibiting immune checkpoints is a promising therapeutic approach to trigger antitumor immune responses and eliminate tumor evasion [34]. Blocking these inhibitory checkpoints can reactivate exhausted T cells, restore their antitumor activity, and promote the elimination of tumor cells [34]. Recently, immunotherapy has been suggested as a viable and promising treatment for a large number of HCC patients [34]. 

### 3.1. PD-1/PD-L1 Inhibitors

PD-1 and its ligand (PD-L1) interaction downregulate T-cell responses by inhibiting their activation and cytotoxicity, preventing immune response and maintaining immune homeostasis [35]. PD-1 expressed on the surface of T cells interacts with PD-L1, which is expressed on the surface of tumor cells or antigen-presenting cells [36]. This interaction inhibits T-cell activation to prevent excessive immune response, resulting in tumor immune escape [36]. Upon the binding of PD-1 to PD-L1, downstream signaling cascades involving the recruitment of phosphatases, such as Src homology region 2-containing protein tyrosine phosphatase-2 (SHP-2), are activated [36]. This activation promotes the dephosphorylation of the signaling molecules that attenuate T-cell receptor signaling, leading to the inhibition of cytokine production and the cytotoxic activity of T cells [36]. Blocking PD-1 and PD-L1 interaction recovers the immune responses of CD8^+^ T cells, which leads to the target and elimination of tumor cells [37]. High PD-L1 expression in both intratumoral or neoplastic inflammatory cells is linked to poor prognosis in HCC patients, indicating the potential of PD-1/PD-L1 signaling as a therapeutic target in immunotherapy [38].

In an initial phase II trial, escalating doses of nivolumab (ICI, anti-PD-1 antibody, 0.1–10 mg/kg) were administered every 14 days to three groups of advanced HCC patients: a control group (those without viral infections), an HBV infection group, and an HCV infection group [39]. The objective response rate (ORR) showed a decrease in tumor size in all three groups during the dose-expansion and dose-escalation phases [39]. Patients who received nivolumab 3 mg/kg per day exhibited ORRs of 20% in the dose-expansion phase and 15% in the dose-escalation phase, respectively [39]. In a phase III trial involving 743 HCC patients, OS was not statistically different between the nivolumab group (16.4 months, n = 371) and the sorafenib (TKI) group (14.7 months, n = 372), indicating similar therapeutic performance for both medications [40]. In another phase III trial, 413 advanced HCC patients who did not respond successfully to sorafenib treatment were randomized to receive either 200 mg of pembrolizumab (ICI, anti-PD-1 antibody) (n = 278) on day 1 of every 21-day cycle for up to 35 cycles, or a placebo (n = 135) [41]. Pembrolizumab treatment improved the median OS to 13.9 months, compared to 10.6 months for the placebo, and decreased the risk of death [41].

### 3.2. CTLA-4 Inhibitors

CTLA-4, an inhibitory protein receptor found on the surface of T cells [42], interacts with its ligands CD80 (B7.1) and CD86 (B7.2), expressed on antigen-presenting cells, to inhibit T-cell activation [43]. The inhibition of this pathway allows B7 molecules on antigen-presenting cells to bind with CD28 on T cells, thereby activating naive CD4^+^ and CD8^+^ T cells [44]. Consequently, the CD80-CTLA-4 interaction inactivates T cells, while the CD80-CD28 interaction activates T cells, resulting in a more potent and sustained immune response against tumor cells [44]. Targeting this signaling is important for cancer treatment, as anti-CTLA-4 monotherapy prevents its binding with B7 molecules, inhibiting the negative stimulatory signal and thus promoting T-cell activation, providing persistent immune restoration and a prolonged survival rate in HCC patients [45].

Tremelimumab, a human IgG2 monoclonal antibody against CTLA-4, was given at a dosage of 15 mg/kg once every 3 months to 21 HCV-associated HCC patients until tumor progression [46]. Response rates were modest at 17%, the median time to progression (TTP) was 6.5 months, and the disease control rate was 76.4% [46]. Tremelimumab treatment showed antiviral and antitumor activity, suppressing immune checkpoints with a favorable therapeutic profile and demonstrating fewer side effects in advanced HCC patients [46]. Another trial administered tremelimumab at doses of 3.5 and 10 mg/kg to 32 advanced HCC patients once every 30 days for six doses, with patients completing RFA on day 36 [47]. Tremelimumab increases the T-cell population and accumulates intratumoral CD8^+^ T cells in the TME, leading to antitumor effects, as demonstrated by an OS of 12.3 months and a median TTP of 7.4 months [47].

## 4. Combinations of ICIs with TKIs or Anti-VEGF Antibodies

TKIs inhibit tyrosine kinases and enzymes that are essential for tumor growth [48]. RTKs are cell surface receptors activated by growth factors, triggering downstream signaling that encourages cell proliferation and survival [48]. Combining PD-1/PD-L1 antibodies with TKIs has shown promising therapeutic effects in various cancers, including advanced-stage HCC [26]. The combined approach not only enhances the antitumor immune responses in T cells, but also disrupts signaling pathways in tumor cells that are essential for tumor growth and survival [49]. The combination of immunotherapies and molecular treatments in clinical trials has demonstrated improved OS compared to monotherapy [49]. Some TKIs (sorafenib, lenvatinib, cabozantinib, and regorafenib) target molecular pathways, including platelet-derived growth factor receptor pathways [50]. Cluster of Differentiation 93 (CD93), a transmembrane protein expressed in stem cells, endothelial cells, and monocytes, has many domains such as an extracellular domain linked to a C-type lectin domain [51]. The interaction of CD93 with its ligand multimerin 2 increases endothelial cell adhesion and migration to promote pathological angiogenesis [52]. Elevated CD93 is associated with immune cell infiltration in tumor tissues, low immunotherapy responses in cancer patients, tumor angiogenesis, poor prognosis, high tumor nodes, and metastasis (TNM) stages in several cancer types [53]. A significant increase in CD93 expression is observed in tumor tissues compared to normal tissues in many cancers, including HCC [52]. Therefore, CD93 plays a pivotal role in the regulation of carcinogenic properties, demonstrating its potential as a biomarker for predicting the prognosis and immune infiltration of different cancer types [53]. Recent clinical research has shown a strong correlation between high CD93 expression and unfavorable immunotherapy outcomes [54]. Additionally, a substantial increase in effector T cells by blocking the CD93 pathway makes tumors more susceptible to immune checkpoint treatment [52]. CD93 appears to be a promising therapeutic target for HCC due to its significant role in promoting pathological angiogenesis, tumor progression, and immune cell infiltration, although there are currently no ongoing clinical trials for HCC.

**Figure 1:**
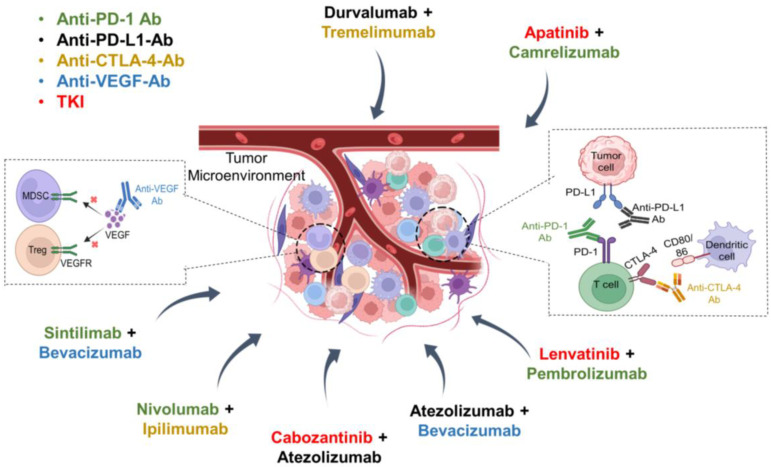
Combination therapy of immune checkpoint inhibitors with tyrosine kinase inhibitors or anti-angiogenic antibodies in HCC. PD-1: programmed death-1; PD-L1: programmed death ligand 1; CTLA-4: cytotoxic T-lymphocyte-associated protein-4; CD80: cluster of differentiation 80; VEGF: vascular endothelial growth factor; MDSC: myeloid-derived suppressor cell; Treg: regulatory T cells; TKI: tyrosine kinase inhibitor.

Anti-VEGF antibodies (bevacizumab and ramucirumab) aim at VEGF signaling, both of which are involved in angiogenesis and tumorigenesis [55]. VEGF is a strong immunomodulatory protein that influences macrophages, myeloid-derived suppressor cells (MDSCs), Treg, and effector T cells to regulate angiogenesis [49]. Anti-VEGF treatment is an effective method that involves the use of anti-VEGF antibodies to target VEGF and its receptors, inhibiting angiogenesis and thereby tumor growth [49]. Clinical studies have suggested that a combined effect of TKI-ICI may provide improved outcomes compared to TKI or ICI monotherapy due to their complementary mechanisms of action in targeting both tumor cell proliferation pathways and immune evasion mechanisms [56]. Anti-PD-1/PD-L1/CTLA-4 antibodies activate effector cells such as DCs, NK cells, and CD8^+^ T cells to promote an antitumor M1 macrophage [57]. TKI, ICI, and anti-VEGF block molecular pathways, immune checkpoints, and VEGF signaling to regulate the TME, as shown in Figure 1. 

A phase Ib trial studied 104 patients with unresectable HCC who received 8 mg of lenvatinib (TKI) + 200 mg of pembrolizumab (ICI, anti-PD-1 antibody) once every 3 weeks for 2 years [58]. The combined use of lenvatinib and pembrolizumab has positive tumor-inhibitory effects and improves survival rate, as demonstrated by a decrease in tumoral Treg, an ORR of 46%, a median OS of 22 months, and a median PFS of 9.5 months [58]. In a different phase 1b trial, advanced-stage HCC patients were divided into two groups: one combination therapy group received atezolizumab (ICI, anti-PD-L1 antibody) 1200 mg with bevacizumab (anti-VEGF antibody) 15 mg/kg, both on the first day of 21-day cycles, while one monotherapy group received atezolizumab alone [59]. This trial reported a median PFS of 5.6 months for the first group compared to 3.4 months for the latter, indicating better efficacy for the combination therapy [59]. A phase I/II trial recruited HCC patients unsuccessfully treated with sorafenib and administered cabozantinib (TKI) 40 mg per day with nivolumab (ICI, anti-PD-1 antibody) 240 mg on the first day of 14-day cycles (doublet immunotherapy), or cabozantinib 40 mg per day with nivolumab 3 mg/kg once every 14 days, along with ipilimumab (ICI, anti-CTLA-4 antibody) 1 mg/kg once after 1.5 months (triplet immunotherapy) [60]. Median OS was 20.1 months and median PFS was 5.1 months for the doublet group, while median OS was 22.1 months and median PFS was 4.3 months for the triplet group, showing promising antitumor efficacy and consistent safety for both combinations [60]. The combined use of TKIs-ICIs (lenvatinib + pembrolizumab, cabozantinib + nivolumab +/− ipilimumab, cabozantinib + atezolizumab, camrelizumab + rivoceranib, camrelizumab + apatinib), or ICIs-ICIs (nivolumab + ipilimumab, durvalumab + tremelimumab), and ICIs-VEGF inhibitors (atezolizumab + bevacizumab, sintilimab + bevacizumab) exhibited great potential for treating advanced HCC by specifically inhibiting angiogenesis and influencing immune cell infiltration, indicating a possibility for improved therapeutic outcomes. 

## 5. Immunotherapeutic Strategies for Different HCC Stages

In general, early-stage patients may have a single lesion larger than 2 cm, or up to three lesions each with a diameter less than 3 cm, but they exhibit a favorable prognosis [61]. Patients in the early stages have preserved liver function and are most appropriate for radical treatment approaches, such as ablation, liver transplantation, and surgical resection [62]. Intermediate-stage HCC involves larger tumors and relatively preserved or compromised liver function [63]. Patients in the advanced-stage have severely impaired liver function and are suitable for palliative approaches, including systemic therapy and chemoembolization, or supportive care [63]. In advanced-stage HCC, tumors are extensively spread, making treatment more challenging and limiting the available therapeutic options compared to early-stage HCC [64]. Standard strategies include chemotherapy and radiation therapy for early-stage HCC; meanwhile, targeted therapy is considered for advanced-stage HCC. However, the efficacy of the targeted therapy is reduced with a higher risk of treatment-related adverse events [65]. Recently, immunotherapy has emerged as a promising approach, offering benefits with fewer procedures associated with side effects from advanced-stage HCC drugs [66]. The combined use of TKIs and ICIs in advanced-stage HCC has shown promising outcomes in terms of tolerability and efficacy [26]. 

### 5.1. Early- or Intermediate-Stage HCC

Neoadjuvant treatments are good for early- or intermediate-stage HCC [67]. These therapeutic approaches, including RFA, TACE, and TARE, are frequently used to shrink tumors before surgery to reduce the risk of tumor spread and improve overall clinical outcomes [68]. A neoadjuvant randomized trial treated nine early-stage HCC patients with nivolumab (ICI, anti-PD-1 antibody) 3 mg on the first day of a 14-day cycle, plus ipilimumab (ICI, anti-CTLA-4 antibody) 1 mg/kg on the first day of every 1.5 months for 2 years [69]. Three out of nine patients showed positive outcomes, with a 33.3% pathological complete response rate and an increase in T-cell infiltration and CD8^+^ T-cell population in the TME facilitating antitumor immune response [69].

A previous pilot study included 30 patients with intermediate-stage HCC who were treated with lenvatinib 8 mg daily, while another group of 60 intermediate-stage HCC patients received TACE [70]. The lenvatinib group had a higher ORR (73% vs. 33%), a prolonged median PFS (16.0 vs. 3.0 months), and a substantially improved OS (37.9 vs. 21.3 months) compared to the TACE group, indicating it had better therapeutic potential for patients with multinodular intermediate-stage HCC [70]. A combination of systemic therapy (immunotherapy and targeted therapy) with local therapy (TACE, TARE, RFA) extended the survival rate of patients with early-stage HCC [71]. A phase II trial evaluated the neoadjuvant therapy of cemiplimab (ICI, anti-PD-1 antibody) 350 mg once every 21 days for two cycles (42 days in total) in 21 resectable early-stage HCC patients. Of these, 20 patients underwent successful surgical resection [72]. A response rate was observed in 15% of the patients, with tumor necrosis occurring in only 20%, while the remaining patients continued to experience tumor progression [72]. This suggests that neoadjuvant cemiplimab alone is not sufficient to induce tumor regression in resectable HCC patients; therefore, more trials are needed to prove the therapeutic efficacy of PD-1 blockades in HCC patients [72]. 

Adjuvant therapies target remaining malignant cells after surgery or other local areas to reduce tumor recurrence and enhance the survival of primary therapy [73]. A randomized phase III trial analyzed 543 HCC patients who received either 200 mg camrelizumab (ICI, anti-PD-1 antibody) on the first day every 14 days plus 250 mg rivoceranib (TKI) once a day, or 400 mg sorafenib (TKI) twice a day [74]. This trial reported a median PFS of 5.3 months and a median OS of 22.1 months for the camrelizumab + rivoceranib group, compared to 3.7 and 15.2 months, respectively, for the sorafenib-only group, indicating that the camrelizumab plus rivoceranib combination therapy improved PFS and OS more effectively than the sorafenib monotherapy [74]. In another randomized trial, 31 intermediate-stage HCC patients who received 400 mg of sorafenib after TACE exhibited a median TTP of 9.2 months without adverse effects, compared to 4.9 months in patients receiving placebo [75]. 

A phase III trial administered atezolizumab (ICI, anti-PD-1 antibody) 1200 mg plus bevacizumab (anti-VEGF antibody) 15 mg/kg once in every 21-day cycle for 12 months to 668 high-risk HCC recurrence patients after ablation or resection [76]. The combination therapy increased recurrence-free survival (RFS) and OS with a longer median follow-up duration compared to active surveillance (without any therapy) [76]. A phase II trial involved 198 early-stage HCC patients who were randomly assigned to receive adjuvant sintilimab (n = 99) once every 21 days for eight cycles or active surveillance (n = 99) [77]. Sintilimab extended median RFS compared to active surveillance (27.7 months vs. 15.5 months) [77]. These trials showed the promising results of neoadjuvant immunotherapies such as nivolumab plus ipilimumab and cemiplimab, with positive results for early- and intermediate-stage HCC. Furthermore, various treatment approaches such as combinations of systemic therapies (camrelizumab + rivoceranib, sorafenib, atezolizumab + bavacizumab, sintilimab) and local therapies (TACE, TARE, RFA) demonstrated higher efficacy with prolonged PFS and OS, improving patient outcomes and treatment success rates.

### 5.2. Advanced-Stage HCC

#### 5.2.1. Monotherapy

The tumor spreads extensively at advanced-stage HCC, leading to poor prognosis and limited treatment approaches compared to the early and intermediate stages [78]. Immunotherapy and combination therapy have gained attention as promising options for treating patients with advanced-stage HCC, as chemotherapy, TACE, and RFA have shown limited efficacy [79]. Nivolumab (ICI, anti-PD-1 antibody) was administered to 145 advanced-stage HCC patients who participated in a phase I/II trial at a dose of 3 mg/kg once every 14 days [80]. This trial demonstrated an ORR of 14.3%, a median response duration of 17 months, and a favorable safety profile [80]. In a phase III trial, 743 patients received nivolumab 240 mg on the first day of every 14-day cycle, or sorafenib 400 mg twice a day [81]. The median OS was 16.4 months for nivolumab, compared to 14.7 months for sorafenib, indicating that nivolumab has high therapeutic activity and a favorable safety profile [81].

A total number of 453 patients with advanced-stage HCC in a phase III trial were administered pembrolizumab (ICI, anti-PD-1 antibody) 200 mg once every 21 days for 35 cycles, or a placebo (no treatment) [82]. The median OS was 14.6 months and PFS was 2.6 months for the pembrolizumab treatment, compared to 13.0 months for OS and 2.3 months for PFS with the placebo [82]. Another phase II trial involved 217 advanced-stage patients who received 3 mg/kg camrelizumab (ICI, anti-PD-1 antibody) once every 14 or 21 days for 2 years [82]. This trial reported a median OS of 14.2 months and an ORR of 14.7% with manageable toxicity [83].

A phase III trial compared tislelizumab (ICI, anti-PD-1 antibody) (200 mg one time every 21 days) to sorafenib (TKI) (400 mg twice a day) as a first-line therapy in 672 patients with unresectable advanced HCC until tumor progression [84]. Tislelizumab exhibited greater potential with a higher ORR (14.3%) and mean OS (15.9 months) and lower PFS (2.2 months) compared to sorafenib (5.4%, 14.1 months, 3.6 months, respectively) [84]. Previous studies have reported that nivolumab [81], pembrolizumab [85], and tislelizumab [84] are effective, indicating promising efficacy and manageable safety over sorafenib monotherapy. Accumulating evidence demonstrates that combination therapy targeting multiple pathways has shown even better results in enhancing OS, improving response rates, and reducing side effects in advanced HCC patients.

#### 5.2.2. Combination Therapy 

In a phase III trial, advanced HCC patients were randomly assigned to receive either cabozantinib (TKI) 40 mg once per day plus atezolizumab (ICI, anti-PD-L1 antibody) 1200 mg on the first day of every 21-day cycle, or sorafenib (TKI) 400 mg twice a day until tumor progression [86]. The cabozantinib plus atezolizumab combination therapy significantly improved the median PFS compared to sorafenib alone (6.8 months vs. 42 months); however, the same trend was not observed in the OS of the two groups [86]. Atezolizumab (ICI, anti-PD-L1 antibody) 1200 mg plus bevacizumab (anti-VEGF antibody) 15 mg/kg once every 21 days were administered to 104 patients with advanced HCC, while 59 patients received bevacizumab only in a phase Ib trial [59]. The median PFS was 5.6 months for atezolizumab plus bevacizumab combination therapy and 3.4 months for the atezolizumab monotherapy [59]. A phase III trial compared patients who received sintilimab (ICI, anti-PD-1 antibody) 200 mg and bevacizumab (anti-VEGF antibody) 15 mg/kg on the first day of every 21-day cycle to those who received sorafenib 400 mg twice a day [87]. The authors found a higher median PFS of 4.6 months for the combination therapy in comparison to 2.8 months for sorafenib monotherapy [87]. 

A nonrandomized phase II trial included advanced-stage HCC patients who received camrelizumab (ICI, anti-PD-1 antibody) 3 mg/kg once every 14 days with a daily dose of apatinib (TKI) 250 mg for 2 years [88]. This trial showed a median PFS of 5.7 months, an ORR of 34%, and a one-year survival rate of 74.7%, indicating the promising efficacy and safety of this combination [88]. In a phase Ib trial, 100 untreated unresectable HCC patients were administered lenvatinib (TKI) 8 mg every day plus pembrolizumab (ICI, anti-PD-1 antibody) 200 mg on the first day of a 21-day cycle for a total of 11 cycles [58]. The results showed an ORR of 46%, a median PFS of 9.3 months, and a median OS of 22 months, suggesting the substantial antitumor efficacy of this combination therapy [58]. However, the authors revealed that 67% of the patients reported adverse events [58]. A phase III trial randomly assigned 794 patients to take either lenvatinib (8 mg/day) with pembrolizumab (200 mg on the first day of every 21 days) or lenvatinib with a placebo [89]. Lenvatinib with pembrolizumab resulted in a median PFS of 8.2 months and a median OS of 21.2 months, whereas the lenvatinib with placebo group reported a median PFS of 8.0 months and a median OS of 19.0 months [89]. 

A randomized trial divided 148 sorafenib-treated HCC patients into three groups [90]. The first group evaluated the effects of combining nivolumab (ICI, anti-PD-1 antibody) 1 mg/kg with ipilimumab (ICI, anti-CTLA-4 antibody) 3 mg/kg for four doses every 21 days, then nivolumab 240 mg every 14 days [90]. The second group was administered with nivolumab 3 mg/kg plus ipilimumab 1 mg/kg once every 21 days for four doses, followed by nivolumab 240 mg every 14 days [90]. The third group received nivolumab 3 mg/kg plus ipilimumab 1 mg/kg [90]. The results showed ORRs of 32% in the first group, 27% in the second, and 29% in the third group, respectively [90]. The first group’s dosage exhibits potential as a therapeutic option for advanced HCC patients and was approved by the FDA based on the findings of this trial [90]. In a phase I/II trial, 332 unresectable HCC patients who received prior sorafenib treatments were randomly assigned into four groups [91]. The first group received a combination of durvalumab (ICI, anti-PD-L1-antibody) 1500 mg and tremelimumab (ICI, anti-CTLA-4-antibody) 300 mg once every 14 or 21 days. The second group was administered durvalumab 1500 mg and tremelimumab 75 mg once every month for four doses [91]. The third and fourth groups were given durvalumab monotherapy (1500 mg one time after 1 month) and tremelimumab monotherapy (750 mg once every month for seven doses followed by one dose after every 3 months) [91]. This trial demonstrated an ORR of 24% and a median OS of 18.7 months in the first group, 9.5% and 11.3 months in the second group, 10.6% and 13.6 months in the third group, and 7.2% and 15.1 months in the fourth group [91]. 

**Figure 2:**
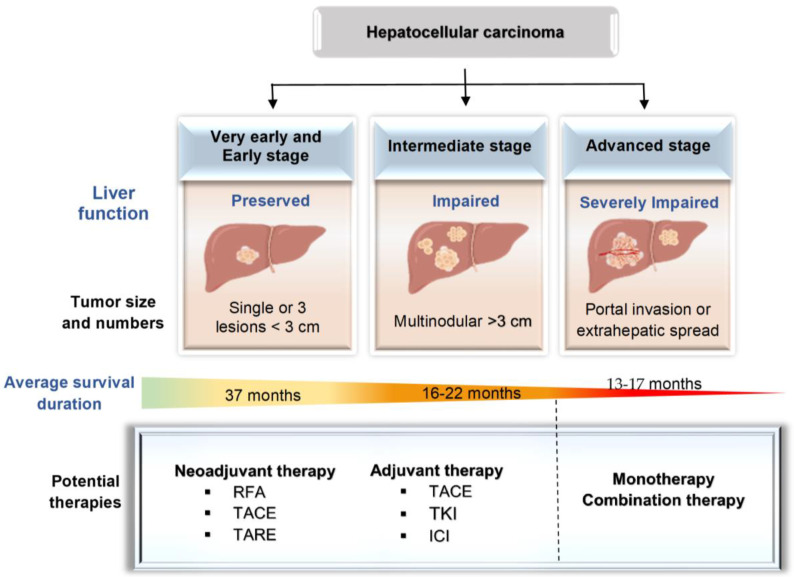
HCC treatment across different stages. The progressive decrease in liver function with an increase in tumor size and number throughout the stages of HCC, from preserved function in early stages to impaired function in intermediate stages, and finally severe dysfunction in advanced stages. Neoadjuvant therapies are used before surgery and ablation to shrink tumor size and remove tumor, while adjuvant therapies are performed after primary treatments to reduce the risk of recurrence in early– or intermediate–stage HCC. Advanced HCC treatment primarily involves systemic therapies, including monotherapy and combination therapy, to enhance survival outcomes. ICIs: immune checkpoint inhibitors; TKIs: tyrosine kinase inhibitors; RFA: radiofrequency ablation; TACE: transarterial chemoembolization; TARE: transarterial radioembolization.

HCC patients (n = 338) at the advanced stage were randomly selected into two groups: lenvatinib treatment (12 mg one time daily) only and lenvatinib plus TACE [92]. The median PFS was 10.6 months and the median OS was 17.8 months for lenvatinib-TACE, while they were 6.4 months and 11.5 months for lenvatinib monotherapy [92]. Therefore, the lenvatinib–TACE combination therapy illustrated higher therapeutic potential compared to lenvatinib monotherapy [92]. A phase III trial randomized 1171 patients to receive tremelimumab (n = 393) 300 mg plus durvalumab 1500 mg once every 30 days, durvalumab (n = 389) 1500 mg monotherapy, or sorafenib (n = 389) 400 mg monotherapy [93]. The results showed that 30.7% of patients in the combination therapy group, 19.8% of patients in the sorafenib group, and 15.9% of patients in the durvalumab group were still alive at 36 months [93]. This demonstrated better long-term OS and a favorable safety profile for the combination therapy compared to the monotherapy [93]. Table 1 summarizes the combination immunotherapies for HCC patients described in this review. Figure 2 summarizes current treatment strategies depending on different HCC stages.

## 6. Alternative Therapeutic Strategies Targeting Immune Cells for HCC

HCC often exhibits challenges in conventional therapies because of drug resistance, tumor heterogeneity, metastasis, and tumor recurrence resulting in the need for therapeutic approaches beyond immunotherapy [95]. DCs can be fused with tumor cell lysates or antigens and present tumor-derived antigens to T cells, leading to immune responses by differentiating and activating antigen-specific CD8^+^ T cells to eradicate HCC [96]. The functions of DCs are impaired by tumor-suppressive microenvironments such as MDSCs, macrophages, and dysregulated signaling pathways in HCC, which promote tumor progression and immune evasion [97]. In a phase II trial, 156 HCC patients were randomly assigned into two groups: the control group (n = 79, no treatment) and the DC transplantation group (n = 77, patients were injected with 3 × 10^7^ DC cells loaded with tumor-associated antigens six times in 14 weeks) [98]. This trial revealed the low risk of recurrence, improved survival rate, and tumor-specific immune responses in patients with DC transplantation [98]. Autologous DC immunization is well tolerated, safe, and produces antigen-directed immune responses, along with evidence of antitumor activity [99]. 

Other trials investigated the efficacy of the transplantation of T cells, instead of DCs, to influence the immune system’s response in targeting tumor cells [100]. A trial randomized 150 patients into two groups, one including 76 patients with adoptive cell transfer and the other comprising 74 patients with no adjuvant therapy [101]. In this trial, the patient’s own T cells were isolated and activated in vitro with CD3 and IL-2 before being transplanted [101]. This approach resulted in a 41% decrease in HCC recurrence risk with substantially higher RFS in the transplanted group compared to the control group [101]. A phase III trial investigated 230 HCC patients receiving adjuvant adaptive cell therapy with activated cytokine-induced killer cells (originated by incubating patient’s peripheral mononuclear cells with anti-CD3 antibody and IL-2) in comparison to a control group (no transplantation) [102]. The results showed extended RFS (44 vs. 30 months), improved OS (3 vs. 12 deaths), and reduced cancer-related deaths (2 vs. 9 deaths) in the immunotherapy group compared to the control group [102]. Out of 94 HCC patients in a nonrandomized trial, 43 patients received adjuvant therapy with a DC vaccine loaded with autologous tumor lysate plus activated T cells after surgery, while 52 patients underwent surgery only [103]. DC plus T-cell patients exhibited improved RFS and a higher median OS compared to those who underwent surgery alone (RFS: 24.5 vs. 12.6 months; median OS: 97.7 vs. 41.0 months) [103]. An administration of DCs and ex-vivo-activated T cells has been demonstrated to prevent recurrence and improve long-term survival rates in HCC patients [103].

Chimeric antigen receptor (CAR) T cells are genetically modified cytotoxic effector T cells that possess specific antigen-recognition properties [104]. Once these T cells recognize antigens expressed on both cancer cells and healthy tissues, they initiate an immune response to eliminate them [104]. In a phase II trial, 21 HCC patients received CART-133 T cells (patient’s own genetically engineered T cells) and showed a median OS of 12 months and a median PFS of 6.8 months [105]. Although CAR T cells have potential as a novel HCC therapy, further trials are required to highlight the promising antitumor activity of CAR T-cell therapy in advanced HCC with manageable adverse events.

## 7. Limitations of Current Immunotherapies

Immunotherapy could present challenges in HCC due to the liver’s complex immunobiology and cause immune-related adverse events, requiring a close assessment of patients and personalized treatment approaches such as genomic sequencing, biomarker analysis, and patient-derived xenografts to find effective drug combinations and dosages [106]. The majority of HCC patients have a complex TME, which consists of many layers of immunosuppressive cells at each stage of the cancer-immunity cycle, leading to insensitivity to TKI and ICI monotherapy [107]. Combination immunotherapies are promising to improve HCC treatment outcomes, particularly for patients who do not respond to monotherapy [108]. Combination immunotherapies present significant limitations and challenges such as liver, bowel, and skin toxicity, as well as collateral damage to other organs by influencing multiple biological pathways [109]. Since the rates of adverse events could be higher in combination therapies compared to monotherapies, carefully designing the duration and doses of combination therapies is crucial to achieve the optimal normalization of the TME in HCC, minimize the incidence of side effects, improve drug delivery, and enhance immune responses [110]. The cost of combination therapies could be significantly higher than monotherapies; therefore, increasing affordability and accessibility for patients is a crucial challenge [111]. 

A higher percentage of HCC patients treated with TKIs alone (46%) experienced serious adverse effects compared to those treated with ICIs (24%) or a combination therapy of TKIs and ICIs (36%), although liver-related toxic effects were similar across all therapies [112]. The most common toxicities are abdominal pain (33%), nausea (47%), hypertension (29%), and fatigue (57%) [113]. The efficacy of immunotherapy may vary among patients, highlighting the need for better biomarkers to predict treatment responses and evaluate personalized therapies [114]. Combination therapies in cancer treatments can improve efficacy but may lead to increased toxicity, severe adverse effects, high costs, and complicated patient management [115]. These challenges require careful observation of the patient’s condition, personalized approaches, and strategies to manage costs and ensure a treatment plan [26]. Effective strategies to reduce toxicity include optimizing drug doses based on patient’s age, weight, tumor size, and hepatic function, utilizing targeted drug delivery systems, and developing novel agents with improved safety profiles by designing drugs that target pathways involved in tumor formation [26].

## 8. Ongoing Clinical Studies

Preclinical and clinical trials are investigating mechanism-based therapies to improve the efficacy of immunotherapy by targeting immunosuppressive pathways to help identify effective therapeutic approaches. Several clinical trials are currently underway to develop novel therapies, suggesting the possibility for further advancements. Nivolumab plus relatlimab (ICI, anti-LAG-3 antibody) and bevacizumab (NCT05337137) (phase I); pembrolizumab plus lenvatinib with belzutifan (HIF-2α inhibitor) (NCT04976634), nivolumab plus ipilimumab (NCT05199285), rulonilimab (ICI, anti-PD-1 antibody) plus lenvatinib (NCT05408221), oxaplatin (chemotherapy drug) plus camrelizumab and apatinib (NCT05412589), durvalumab plus tremelimumab with Y-90 SIRT (NCT04522544) (phase II); and regorafenib plus pembrolizumab vs. TACE/TARE (NCT04777851), atezolizumab plus bevacizumab with or without tiragolumab (ICI, anti-TIGIT antibody) (NCT05904886), and durvalumab plus tremelimumab +/− lenvatinib with TACE (NCT05301842) (phase III) are currently undergoing clinical trials for HCC patients. These combination therapies may be designed and developed with different mechanisms of action, focusing particularly on specific targets and an improved safety profile to enhance PFS and OS for advanced-stage HCC. Future treatments may involve triplet combination therapies or biomarker-based approaches for the targeted patient population. Table 2 summarizes the combination immunotherapies in ongoing clinical trials for HCC patients described in this review.

## 9. Conclusions and Future Perspectives

Current studies and clinical trials demonstrate the promising therapeutic effects of combination therapies against HCC, offering potential strategies to reduce resistance mechanisms and improve patient outcomes. Resistance to immunotherapies in some HCC patients is influenced by factors such as tumor heterogeneity, immune escape mechanisms, and adaptive resistance. Strategies to overcome these challenges and reduce these risks include combination therapies, personalized medicine based on tumor profiles, TME modulation, the management of genetic/epigenetic factors, immune response enhancement, and continuous patient surveillance in adaptive clinical trials [116,117]. Understanding the specific conditions of each patient is crucial to adapting therapies and avoiding resistance in HCC treatment. Monotherapies and combination therapies using TKIs and/or ICIs have promising therapeutic effects, specifically in targeting tumor cells for the management of advanced-stage HCC. Patients who received anti-VEGF antibodies/TKIs with anti-PD-1/PD-L1/CTLA-4 antibodies showed better ORR, OS, and PFS than those who received TKI or ICI monotherapy. Combination therapy (ICIs-ICIs, TKIs-ICIs, anti-VEGF-ICIs) is an active area of research aimed at improving therapeutic effects by targeting the TME and the multiple pathways involved in tumor growth, enhancing immune response, and reducing adverse effects. Regulatory challenges including, but not limited to, the safety and effectiveness of multiple drugs, unclear endpoints, and the need for specialized regulatory pathways can delay the approval of combination therapies for HCC and impact treatment availability. 

The monotherapies of nivolumab and pembrolizumab, as well as the combinations of lenvatinib and pembrolizumab, nivolumab and ipilimumab, and durvalumab and tremelimumab, showed promising efficacy with better median OS and PFS duration in the treatment of advanced HCC. Therefore, the combination of nivolumab or pembrolizumab plus tremelimumab +/− bevacizumab could demonstrate excellent performance for HCC therapies, although no clinical trials have been conducted for this combination. Combination therapy in HCC treatment offers potential benefits, but also poses several disadvantages and limitations, including high cost, increased toxicity, higher risk of drug resistance, treatment-related adverse events, diverse patient responses, and severe collateral damage in different organs. Potential solutions to address these shortcomings include combination therapies targeting multiple pathways, personalized treatment approaches based on genomic profiling, and the development of novel agents targeting resistance mechanisms, such as tumor heterogeneity and adaptive signaling pathways. Furthermore, the use of combination therapies for HCC also presents significant financial challenges due to the high cost of multiple drugs, and may be limited globally by variations in healthcare, infrastructures, and policies. The high cost of immunotherapy could be alleviated by implementing a large-scale production of specific TKIs/ICIs, discovering new affordable drugs, focusing on cost-effective strategies, emphasizing predictive biomarkers, and exploring herbal and traditional medicines. Establishing a benchmark method is crucial because it can lower costs, reduce adverse effects, and improve overall treatment accessibility and effectiveness.

Future studies may focus on specific drug combinations and triplet therapies that show the most promising efficacy and safety in HCC. In addition, identifying predictive biomarkers and validating their efficacy plays an important role in evaluating immunotherapy in HCC patients and enabling more personalized treatment approaches. Ongoing progress is necessary to enhance, validate, and integrate these biomarkers into routine clinical practice. Further research, including clinical trials and long-term follow-up studies, is needed to evaluate the safety performance of immunotherapies and combination therapies, analyze the clinical profiles of HCC patients, and determine the most effective personalized treatment strategies. In conclusion, combination therapies hold great potential for HCC treatment, but additional clinical studies are necessary to enhance their efficacy, safety, and affordability.

## Figures and Tables

**Table 1:** Efficacy and safety of various combinations of immunotherapy and targeted therapy in selected HCC clinical trials.

Trial ID	Treatment	Trial Phase	No. of Patients	Drug Dose	Primary Outcomes at the End Point			Reference
					ORR	PFS	OS	
NCT03713593	Lenvatinib + pembrolizumab	II	100	Lenvatinib 8 mg orally every day + pembrolizumab 200 mg intravenously (IV) on the first day of a 21-day cycle.	46%	9.3 months	22 months	[58]
NCT01658878	Cabozantinib + nivolumab (doublet) +/− ipilimumab (triplet)	I/II	36 (doublet) and 35 (triplet)	Cabozantinib 40 mg orally per day with nivolumab 240 mg IV once in 14 days vs. cabozantinib 40 mg per day + nivolumab 3 mg/kg once after 14 days with ipilimumab 1 mg/kg IV once in 1.5 months	17% vs. 29%	5.1 months vs. 4.3 months	20.1 months vs. 22.1 months	[60]
NCT04102098	Atezolizumab + bevacizumab vs. sorafenib	III	501	Atezolizumab 1200 mg + bevacizumab 15 mg/kg IV on day one after every 21-day cycle	27% vs. 12%	6.8 months vs. 4.3 months	19.2 months vs. 13.4 months	[94]
NCT01658878	Nivolumab + ipilimumab	II	148	Nivolumab 1 mg/kg + ipilimumab 3 mg/kg IV once after every 21 days	32%	N/A	22.8 months	[90]
NCT02519348	Durvalumab + tremelimumab	I–II	332	Durvalumab 1500 mg + tremelimumab 300 mg IV once every 30 days	95%	N/A	18.7 months	[91]
NCT03755791	Cabozantinib + atezolizumab vs. sorafenib	III	837	Cabozantinib 40 mg orally once a day + atezolizumab 1200 mg IV every 21 days	N/A	6.8 months vs. 4.2 months	15.4 months vs. 15.5 months	[86]
NCT03794440	Sintilimab + bevacizumab vs. sorafenib	II-III	595	Sintilimab 200 mg + bevacizumab 15 mg/kg IV on day 1 of every 21 days vs. sorafenib 400 mg orally twice daily	25·0%	4.6 months vs. 2.8 months	Longer OS for combination therapy compared to sorafenib monotherapy	[87]
NCT03905967	Lenvatinib + chemoembolization vs. lenvatinib	III	338	Lenvatinib 8 mg orally once daily for patients < 60 kg, 12 mg for ≥ 60 kg	54.1% vs. 25.0%	10.6 months vs. 6.4 months	17.8 months vs. 11.5 months	[92]
NCT02576509	Nivolumab vs. sorafenib	III	743	Nivolumab 240 mg IV once after every 14 days vs. sorafenib 400 mg orally twice daily	15% vs. 7%	5.0 months vs. 4.0 months	16·4 months vs. 14·7 months	[81]
NCT03062358	Pembrolizumab vs. placebo	III	453	Pembrolizumab 200 mg IV once every 21 days	12.7% vs. 1.3%	2.6 months vs. 2.3 months	14.6 months vs. 13 months	[82]
NCT03412773	Tislelizumab vs. sorafenib	III	672	Tislelizumab 200 mg IV on day 1 of every 21-day cycle or sorafenib 400 mg twice a day	14.3% vs. 5.4%	2.2 months and 3.6 months	15.9 months vs. 14.1 months	[84]
NCT03764293	Camrelizumab + rivoceranib	III	543	Camrelizumab 200 mg IV on day 1 of every 14 days + rivoceranib 250 mg orally once a day	N/A	5.3 months	22.1 months	[74]
NCT03298451	Tremelimumab + durvalumab vs. sorafenib	III1	1171	Tremelimumab 300 mg + durvalumab 1500 mg IV on day 1 of every 14 days	N/A	N/A	36-month OS was 30.7% for combination therapy and 19.8% for sorafenib monotherapy	[93]
NCT03463876	Camrelizumab + apatinib	II	120	Camrelizumab 3 mg/kg IV on day 1 of every 14 days + daily oral dose of apatinib 250 mg	34.3%	5.7 months	N/A	[88]
NCT03713593	Lenvatinib + pembrolizumab	III	794	Lenvatinib 8 mg/day orally with pembrolizumab 200 mg IV on day 1 of every 21 days	N/A	8.2 months	21.2 months	[89]

**Table 2:** Ongoing clinical trials evaluating the efficacy of various drug combinations for the treatment of HCC. These trials are ongoing, and no associated publications are available for these studies.

Trial ID	Treatment	Trial Phase	No. of Patients (Estimated)	Current Status
NCT04976634	Pembrolizumab + lenvatinib with belzutifan	II	730	Ongoing
NCT05199285	Nivolumab + ipilimumab	II	40	Ongoing
NCT05408221	Rulonilimab + lenvatinib	II	576	Ongoing
NCT05412589	Oxaplatin + camrelizumab and apatinib	II	35	Ongoing
NCT04777851	Regorafenib + pembrolizumab vs. TACE/TARE	III	496	Ongoing
NCT05337137	Nivolumab + relatlimab and bevacizumab	I	162	Ongoing
NCT04522544	Durvalumab + tremelimumab with Y-90 SIRT	II	55	Ongoing
NCT05904886	Atezolizumab + bevacizumab with or without tiragolumab	III	650	Ongoing
NCT05301842	Durvalumab + tremelimumab +/− lenvatinib with TACE	III	725	Ongoing

## Data Availability

Not applicable.
